# The Operations of Surgery

**Published:** 1897-09

**Authors:** 


**The Operations of Surgery.** By W. H. A. Jacobson, M.Ch. Third Edition. Pp. xiv., 1337. London: J. & A. Churchill. 1897.

We are glad that Mr. Jacobson has resisted the temptation to expand this book into two volumes. Although it has been considerably enlarged, and although the number of illustrations has been doubled since the first edition, yet by the employment of a deeper page, and the occasional use of small print in describing cases and in the accounts of the less frequently employed operations, he has kept the book within the compass of a single volume. We do not know whether most to admire the exceeding clearness and lucidity of the descriptions of the various operations in this book, or the beauty of the illustrations by which they are accompanied; both together form a delightful volume which may well make the student of a former generation envious of one of the present day. We know no work on the subject of operative surgery at all equal to this one, and we can strongly recommend it to all students.

More than one-third of the whole volume is devoted to the subject of abdominal surgery; and it is here more than in any other part of the book that we notice the care taken by the author to keep abreast with recent work. No less than ten operations for the radical cure of hernia by the open method are given and fully described, and of these the author (we think rightly) prefers Macewen's, but here, as elsewhere in the book, he displays eminent fairness, and is always ready to point out the advantages claimed by introducers for their own operations.

Paul's tubes for artificial openings in the intestine are described and illustrated, as are also Murphy's buttons, Mayo Robson's bobbins, and Senn's decalcified bone plates. Greig Smith's operation for the radical cure of umbilical hernia is described with approval, and his drawings are reproduced from Walsham's *Surgery*. In operations for the removal of the tongue the methods of Whitehead, Syme, and Kocher are described, and also the method of removing by the écraseur. In Whitehead's operation Mr. Jacobson approves of a preliminary laryngotomy with plugging of the back of the mouth, although he acknowledges that the introducer of this method never makes use of these preliminaries. Kocher's method is not spoken of with much approval. In removal of the mammary gland for cancer Mr. Jacobson advocates removal of the whole organ and clearing out the axilla in every case, following the advice of Mr. Banks rather than that of Mr. Butlin, although he would not go so far as Professor Halsted, who would remove the whole thickness of the pectoralis major and divide the pectoralis minor. We think here, as in many other cases, Mr. Jacobson adopts an eminently judicious tone, and gives us sound advice, which may be safely followed by young surgeons who seek in this volume for a trustworthy guide.

